# Complete plastome sequence of *Piper laetispicum* (Piperaceae): An endemic plant species in South China

**DOI:** 10.1080/23802359.2018.1511850

**Published:** 2018-10-27

**Authors:** Ming-Tao Wang, Jian-Hua Wang, Kun-Kun Zhao, Zhi-Xin Zhu, Hua-Feng Wang

**Affiliations:** Hainan Key Laboratory for Sustainable Utilization of Tropical Bioresources, Institute of Tropical Agriculture and Forestry, Hainan University, Haikou, China

**Keywords:** *Piper laetispicum*, plastome, phylogeny, genome structure, Piperaceae

## Abstract

*Piper laetispicum* is dioecious climbers woody with 10 m tall, which is an endemic species in China. It grows on trees or rocks in forests (100–600 m) in Guangdong and Hainan Province of China. Here, we report and characterize the complete plastid genome sequence of *P. laetispicum* in an effort to provide genomic resources useful for promoting its conservation. The complete plastome is 161,721 bp in length and contains the typical structure and gene content of angiosperm plastome, including two inverted repeat (IR) regions of 27,125 bp, a large single copy (LSC) region of 89,224 bp and a small single copy (SSC) region of 18,247 bp. The plastome contains 114 genes, consisting of 80 unique protein-coding genes, 30 unique tRNA gene, and four unique rRNA genes. The overall A/T content in the plastome of *P. laetispicum* is 61.70%. The complete plastome sequence of *P. laetispicum* will provide a useful resource for the conservation genetics of this species as well as for the phylogenetic studies for *Piper*.

*Piper laetispicum* C. DC (Piperaceae, Piperales) is dioecious climbers woody with 10 m tall, which is an endemic species in China. It grows on trees or rocks in forests (100–600 m) in Guangdong and Hanan Province of China (Cheng et al. 1999). Consequently, the genetic and genomic information is urgently needed to promote its systematics research and the development of conservation value of *P. laetispicum*. Here, we report and characterize the complete plastome of *P. laetispicum* (GenBank accession number: MH678665, this study). This is the first report of a complete plastome for the *P. laetispicum*.

In this study, *P. laetispicum* was sampled from Diaoluo Mountain (18.67°N, 109.88°E), which is a National Nature Reserve of Hainan, China. A voucher specimen (H.F. Wang, B36) was deposited in the Herbarium of the Institute of Tropical Agriculture and Forestry (HUTB), Hainan University, Haikou, China.

The modified cetyltrimethyl ammonium bromide (CTAB) protocol of Doyle and Doyle (1987) was used to extract genomic DNA from dry leaf tissues. The genomic DNA of each sample was quantified and analysed with Agilent 2100 BioAnalyzer (Palo Alto, CA). Samples of yield at least 0.8 μg DNA were selected for subsequent libraries construction and *de novo* sequencing. Genomic DNA of selected samples were used to build the paired-end libraries with 200–400 bp insert size. Libraries were sequenced using BGISEQ-500 platform at BGI (Shenzhen, China) and produced about 8 Gb high quality per sample with 100 bp paired-end reads. Raw reads were trimmed using SOAPfilter_v2.2 with the following criteria (1) reads with >10% base of N; (2) reads with >40% of low quality (value ≤10); (3) reads contaminated by adaptor and produced by PCR duplication. Around 6 Gb clean data were assembled against the plastome of *Piper kadsura* (KT223569.1) (Lee et al. 2015) using MITO bim v1.8 (Hahn et al. 2013).

The plastome was annotated using Geneious R8.0.2 (Biomatters Ltd., Auckland, New Zealand) against the plastome of *Piper kadsura* (KT223569.1). The annotation was corrected with DOGMA (Wyman et al. 2004).

The plastome of *P. laetispicum* was found to possess a total length 161,721 bp with the typical quadripartite structure of angiosperms, containing two inverted repeats (IRs) of 27,125 bp, a large single copy (LSC) region of 89,224 bp and a small single copy (SSC) region of 18,247 bp. The plastome contains 114 genes, consisting of 80 unique protein-coding genes, 30 unique tRNA genes, and four unique rRNA genes. Among these genes, 15 genes (*trnK-UUU, rps16, trnG-UCC, atpF, rpoC1,trnL-UAA, trnV-UAC, trnI-GAU, trnA-UGC, rpl12, petB, petD, rpl16, ndhB, ndhA*) possessed a single intron and three genes (*ycf3, clpP, rps12*) had two introns. The gene *rps12* was found to be trans-spliced, as is typical of angiosperms. The overall A/T content in the plastome of *P. laetispicum* is 61.70%, in which the corresponding value of the LSC, SSC, and IR region was 63.20%, 67.90%, and 57.10%, respectively.

We used RAxML (Stamatakis 2006) with 1000 bootstraps under the GTRGAMMAI substitution model to reconstruct a maximum likelihood (ML) phylogeny of seven published complete plastome of Piperales and one published complete plastome of Canellales, using *Magnolia alba* (Magnoliaceae, Magnoliales) and *Calycanthus chinensis* (Calycanthaceae, Laurales) as outgroups. The phylogenetic analysis indicated that *P. laetispicum* is closer to *P. kadsura* with maximum bootstrap support (Figure 1). Most nodes in the plastome ML trees were strongly supported. The complete plastome sequence of *P. laetispicum* will provide a useful resource for the conservation genetics of this species as well as for the phylogenetic studies for *Piper*.

**Figure 1.**
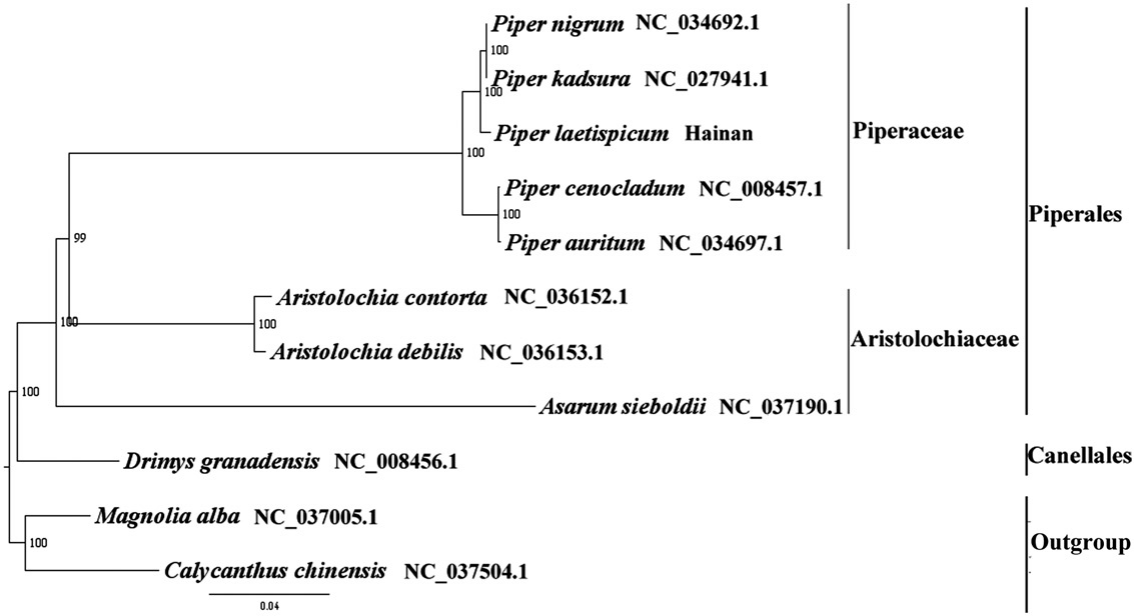
The best ML phylogeny recovered from 11 complete plastome sequences by RAxML. Accession numbers: *Piper laetispicum* (MH678665, this study), *Piper nigrum* NC_034692.1, *Piper kadsura* NC_027941.1, *Piper cenocladum* NC_008457.1, *Piper auritum* NC_034697.1, *Aristolochia contorta* NC_036152.1, *Aristolochia debilis* NC_036153.1, *Asarum sieboldii* NC_037190.1, *Drimys granadensis* NC_008456.1; outgroup: *Magnolia alba* NC_037005.1, *Calycanthus chinensis* NC_037504.1.
